# Upregulation of MiR-205 under hypoxia promotes epithelial–mesenchymal transition by targeting ASPP2

**DOI:** 10.1038/cddis.2016.412

**Published:** 2016-12-08

**Authors:** Xingwen Wang, Miao Yu, Kunming Zhao, Mengmeng He, Wenjie Ge, Yuhui Sun, Yihua Wang, Haizhu Sun, Ying Hu

**Affiliations:** 1 School of Life Science and Technology, Harbin Institute of Technology, Shenzhen, China; 2 Shenzhen Graduate School of Harbin Institute of Technology, Shenzhen, China; 3 School of Chemical Engineering and Technology, Harbin Institute of Technology, Harbin, China; 4 The First Affiliated Hospital, Harbin Medical University, Harbin, China; 5 Biological Sciences, Faculty of Natural and Environmental Sciences, University of Southampton, Southampton, UK; 6 The Second Affiliated Hospital, Harbin Medical University, Harbin, China

## Abstract

The epithelial–mesenchymal transition (EMT) is one of the crucial procedures for cancer invasion and distal metastasis. Despite undergoing intensive studies, the mechanisms underlying EMT remain to be completely elucidated. Here, we identified that apoptosis-stimulating protein of p53-2 (ASPP2) is a novel target of MiR-205 in various cancers. Interestingly, the binding site of MiR-205 at the 3′-untranslated region of ASPP2 was highly conserved among different species. An inverse correlation between MiR-205 and ASPP2 was further observed *in vivo* in cervical cancers, suggesting MiR-205 may be an important physiological inhibitor of ASPP2. Hypoxia is a hallmark of solid tumor microenvironment and one of such conditions to induce EMT. Notably, MiR-205 was remarkably induced by hypoxia in cervical and lung cancer cells. A marked suppression of ASPP2 was observed simultaneously. Further studies confirmed that hypoxia-induced ASPP2 suppression was mainly attributed to the elevated MiR-205. Interestingly, the alteration of MiR-205/ASPP2 under hypoxia was accompanied with the decreased epithelial marker E-cadherin and increased mesenchymal marker Vimentin, as well as a morphological transition from the typical cobblestone-like appearance to the mesenchymal-like structure. More importantly, MiR-205 mimics or ASPP2 silencing similarly promoted EMT process. By contrast, ASPP2 recovery or MiR-205 inhibitor reversed MiR-205-dependent EMT. Further studies demonstrated that the newly revealed MiR-205/ASPP2 axis promoted cell migration and also increased cell proliferation both *in vivo* and *in vitro*. These data together implicated a critical impact of MiR-205/ASPP2 on promoting EMT. MiR-205/ASPP2 may be potential diagnostic and therapeutic biomarkers in cervical and lung cancers.

The epithelial–mesenchymal transition (EMT) was initially identified as a fundamental tissue remodeling mechanism of embryonic development.^1^ More recently, mounting evidence has suggested that EMT is also one of critical procedures for cancer metastasis.^2, 3, 4, 5^ Given that >90% of cancer-related mortality is due to cancer metastasis, dissecting the precise mechanisms of EMT will facilitate the discovery of novel diagnostic and therapeutic biomarkers and ultimately improve cancer prognosis in general.

Cells undergoing EMT are featured by the loss of cell–cell adhesion and the acquirement of a mesenchymal phenotype, which is accompanied by the typical molecular changes, such as the loss of epithelial marker E-cadherin and the gain of mesenchymal markers Vimentin.^6^ As such, in the process of EMT, epithelial cells will go through fundamental morphological changes from typical cobblestone-like structure to spindle-shaped phenotype with increased cell motilities.^7^ Mechanistically, EMT can be regulated at different levels. The activation of transcriptional factors, such as HIF-1a, snail, twist, slug and *β*-catenin, can modulate EMT process by stimulation or repression panels of EMT-related target genes.^8, 9^ Recently, MicroRNAs (MiRNAs) have also been proved to modulate EMT progress at epigenetic levels.^10, 11^ MiRNAs is found to be endogenously expressed small non-coding RNA gene products of approximately 22 nucleotides that downregulate gene expression by binding to the 3′-untranslated regions (3′-UTRs) of specific target messenger RNAs (mRNAs), leading to mRNA degradation or inhibition of translation.^12^ Despite being discovered not long time ago, MiRNAs' importance in controlling gene transcriptome has gained great attention. It is thus not surprising to know that MiRNAs are important component of EMT machineries. For instance, loss of MiR-200 family or MiR-205 are association with cancer metastasis.^13, 14, 15^ This, at least in part, attributes to its inhibitory activity toward EMT by targeting transcriptional repressors of E-cadherin, ZEB1 and ZEB2.^14, 16, 17^ However, MiRNAs can target various genes and single gene can be modulated by many MiRNAs, therefore, the biological outcomes of MiRNA activation may change with cell content and stimulus, which provides potential explanations for the complicated and even controversial functions of MiRNA. MiR-205 is such a 'double-edged sword' in cancer. Whereas it was initially considered as a tumor suppressor and the majority of its targets reported so far are oncogenes,^18^ recently, its pro-proliferation and pro-metastatic oncogene functions have also been discovered.^19, 20, 21, 22^ However, the underlying mechanisms of this oncogenetic function, particularly in regulating EMT, remain to be elucidated.

ASPP2 belongs to the apoptosis-stimulating proteins of p53 (ASPP) family, which directly interacts with p53 family members and selectively promotes their transcriptional activities toward pro-apoptosis genes.^23, 24^ Further evidences from mice model revealed that ASPP2 is a haploinsufficient tumor suppressor.^25^ Indeed, ASPP2 expression is deregulated in several human tumor types, and ASPP2 suppression is associated with more aggressive phenotypes and poor clinical outcomes of cancers.^26, 27, 28, 29^ Recently, emerging evidence has suggested that ASPP2's functions do not always rely on p53. Mak *et al.*^27^ found that ASPP2 can inhibit cell migration through modulating Src tyrosine kinase activities. In addition, ASPP2 has been reported to form complex with tight junction competent par-3 and regulates cell polarity of neoepithelial cells in a p53-independent manner.^30, 31, 32^ Moreover, it has been recently found that ASPP2 can inhibit EMT by preventing the activation of Wnt signaling in cancers.^33^ These data provide important molecular explanations for the clinical significance of ASPP2 in cancer metastasis. Nevertheless, compared with the biological functions of ASPP2, much less is known about its regulatory mechanisms on gene expression. Underlying mechanisms of ASPP2 regulated by MiRNAs and the resulting biological impacts of such regulation on EMT remains largely unknown.

In this study, our results showed here that ASPP2 is a representative tumor-suppressor target of MiR-205 in multiple cancers. The inverse correlation between ASPP2 and MiR-205 was further revealed *in vivo* in human cervical specimens. Notably, hypoxia is one of the hallmarks of solid tumor microenvironment and also one of such conditions to induce EMT. The newly identified MiR-205/ASPP2 axis was induced by hypoxia exposure and had crucial roles in regulating hypoxia-induced EMT via influencing E-cadherin and Vimentin. Further studies showed that MiR-205/ASPP2 axis contributes to such biological outcomes as the promoted migration and cell survival.

## Results

### MiR-205 is negatively associated with ASPP2 both *in vitro* and *in vivo*

**Figure 1:**
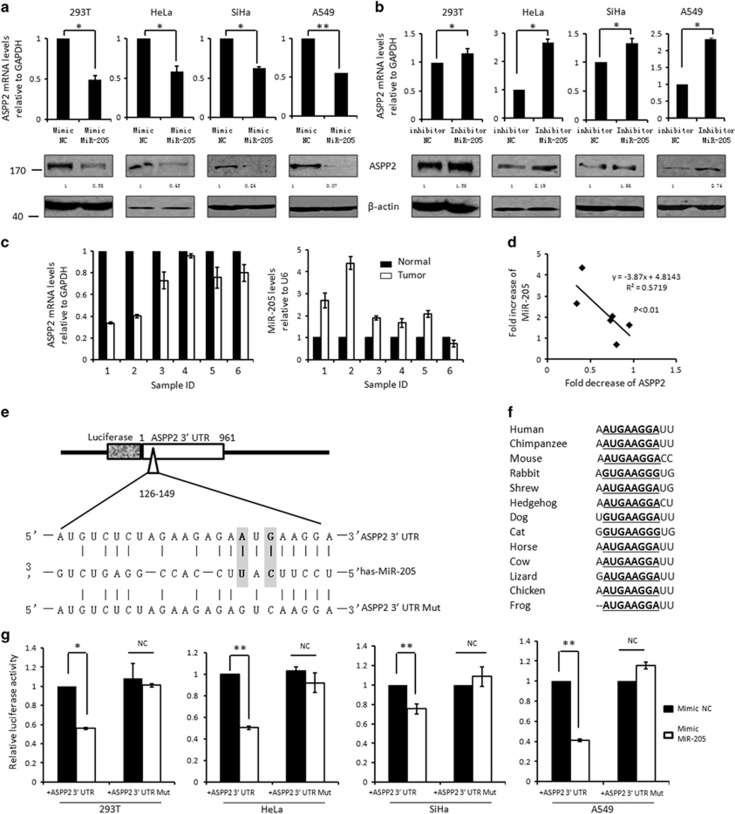
ASPP2 is a direct target of MiR-205. (**a** and **b**) Real-time RT-PCR and western blotting (WB) analysis of ASPP2 at 72 h post-transfection with either MiR-205 mimics/negative controls (NC) mimics (**a**) or MiR-205 inhibitors/NC inhibitors (**b**). *β*-Actin was used as loading controls in WB assays. (**c**) Real-time RT-PCR analysis of ASPP2 and MiR-205 expression in six cervical cancers and the paired normal controls. (**d**) The expression of MiR-205 and ASPP2 were displayed negative linear correlation in cervical cancers. (**e**) Schematic description of the hypothetical duplexes formed by the interactions between the binding site in the ASPP2 3′-UTR (top), MiR-205 (middle) and the mutated ASPP2 3′-UTR (bottom). The seed recognition site is denoted. (**f**) All nucleotides of ASPP2 3′-UTR region that binds with MiR-205 are highly conserved across species as predicated by TargetScan (http://www.targetscan.org/vert_71/). (**g**) The luciferase activities of wild type or mutant 3′-UTR ASPP2 luciferase reporter were measured after transfection with NC mimics or MiR-205 mimics. All histograms represent the mean±S.E.M. from three independent assays. *P<0.05; **P<0.01

In order to understand the underlying epigenetic regulation of ASPP2 by miRNAs, the potential miRNA-binding sites located at 3′-UTR regions of ASPP2 were predicted by the commonly cited bioinformatic tools such as microRNA.org, TargetScan4, miRBase, PicTar and miRanda. Five targets (MiR-205, 221, 222, 144 and 139) were predicted by at least four of above databases (Supplementary Figure S1a). To validate the bioinformatic results, these miRNA mimics were overexpressed respectively in 293T cells, and both mRNA and protein levels of ASPP2 were examined subsequently. MiR-221, 222, 144 and 139 showed no obvious impact on ASPP2's expression at either mRNA or protein level (Supplementary Figure S1b). However, upon MiR-205 mimics treatment, ASPP2 expression was significantly repressed up to 44.9% at mRNA level and up to 35.0% at protein level (*P*<0.01, Supplementary Figure S1b). In addition, the regulation of ASPP2 by MiR-205 is likely a common event, because similar results were obtained in the cervical (Hela and SiHa), lung (A549) and renal (RC-1) cancer cells (Figure 1a, Supplementary Figures S1c and S2). In support of this, MiR-205 inhibitor promoted ASPP2's expression at both mRNA levels and protein levels in all cell models involved (Figure 1b and Supplementary Figure S1c). More impotently, the regulation of ASPP2 by MiR-205 likely also occurs *in vivo*. As shown in Figure 1c, ASPP2 was decreased and MiR-205 was increased in cervical cancers in compared with paired normal controls. Furthermore, the levels of ASPP2 and MiR-205 were inversely correlated with each other, suggesting MiR-205-mediated gene silencing may be one of major mechanisms to inhibit ASPP2 *in vivo* at least in cervical cancers (*r*^2^=0.55, *P*<0.01, Figure 1d).

### ASPP2 is a direct target of MiR-205

According to the bioinformatics analysis, there is one single potential binding sites of MiR-205 at ASPP2 3′-UTR. The complementarity of ASPP2 3′-UTR with the seed sequence of MiR-205 were shown in Figure 1e. Interestingly, the MiR-205-binding site in ASPP2 was conserved among different species (Figure 1f), suggesting that the regulation may have important roles in modulating fundamental functions of ASPP2. We then constructed luciferase reporter containing whole ASPP2 3′-UTR (WT, 961 bp) (Figure 1e), which was co-transfected with the Renilla luciferase reporter plasmid into cells. Remarkably, ASPP2 3′-UTR (WT) luciferase signals were reduced by 50–60% in response to MiR-205 expression in comparison with cells transfected with the same amount of scramble control in 293T (56.0%, *P*<0.05), Hela (50.5%, *P*<0.01), SiHa (75.4%, *P*<0.01) and A549 (41.3%, *P*<0.01) cells (Figure 1g). To further confirm the reduced luciferase signal is due to a direct interaction of MiR-205 and the putative sites at ASPP2 3′-UTR, a MiR-205-binding sites mutated 3′-UTR luciferase reporter, ASPP2 3′-UTR (MT), were generated by mutagenesis kits (Figure 1e). As expected, MiR-205 failed to affect the luciferase signals of ASPP2 3′-UTR (MT) under the same conditions (Figure 1g). These data together suggests that ASPP2 indeed is a direct target of MiR-205.

### ASPP2 is subject to the regulation of hypoxia and confers hypoxia-induced EMT

**Figure 2:**
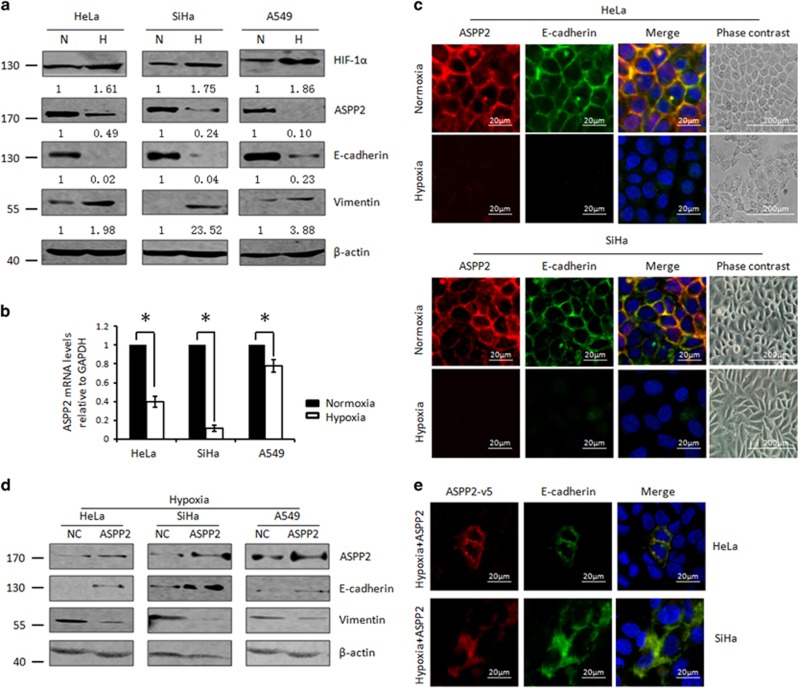
Hypoxia-mediated ASPP2 suppression promotes EMT. (**a**) WB analysis of HIF-1a, ASPP2, E-cadherin and Vimentin expression under normoxia (N) and hypoxia (H) conditions. Protein expression as normalized to the loading control *β*-actin was quantified by Image J software. (**b**) Real-time RT-PCR analysis of ASPP2 expression under normoxia (N) and hypoxia (H) conditions. Histograms represent the mean±S.E.M. from three independent assays. *P<0.05. (**c**) Immunostaining analysis of ASPP2/E-cadherin expression and localization under normoxia and hypoxia conditions. (**d**) WB analysis of ASPP2, E-cadherin and Vimentin after ectopic expression of ASPP2 under hypoxia conditions. (**e**) Immunostaining analysis of E-cadherin and exogenous ASPP2-v5 expression and localization under the conditions described in (**d**)

Hypoxia is a key microenvironmental factor to promote cancer metastasis. As a newly identified tumor suppressor, ASPP2 deregulation has been associated with advanced cancers. We therefore asked whether ASPP2 is subjected to the regulation of hypoxia. As reported previously, we found that HIF-1a was markedly increased with hypoxia (2.0% O_2_) treatment (top panel, Figure 2a). The mRNA levels of its well-known transcriptional target VEGF were also correspondingly increased (Supplementary Figure S3). Under such conditions, ASPP2 was remarkably and consistently decreased at mRNA levels to different extends in cervical (Hela and SiHa) and lung (A549) cancers (*P*<0.05, Figure 2b). In addition, an obvious suppression of ASPP2 protein was also confirmed by both western blotting and immunofluorescence assay as shown in Figures 2a and c, respectively.

Interestingly, ASPP2 was found to locate mainly at cell–cell junction, where it colocalized with E-cadherin under the normoxia conditions (Figure 2c). Upon hypoxia switch, ASPP2 suppression was paralleled with the inhibition of epithelial marker E-cadherin and the induction of mesencymal marker Vimentin (Figures 2a, c and Supplementary Figure S4), suggesting cells were undergoing EMT process. In support of it, cells cultured under the hypoxia condition lost typical epithelial cobblestone-like structure and showed as a more elongated spindle-shaped morphology (Figure 2c). Importantly, when ASPP2 expression was arbitrarily resumed by introducing ASPP2 constructs, the decreased E-cadherin and increased Vimentin were largely restored (Figures 2d and e)**.** In addition, an obvious junctional recruitment of E-cadherin and ASPP2 were observed particularly between two adjacent cells both with exogenous ASPP2 expression under hypoxia conditions (Figure 2e). These data together pointed to the notion that ASPP2 prevents hypoxia-induced EMT and this ability of ASPP2 may be dependent on its precise junctional localization.

### Enhanced MiR-205 promotes EMT via targeting ASPP2 under hypoxia conditions

**Figure 3:**
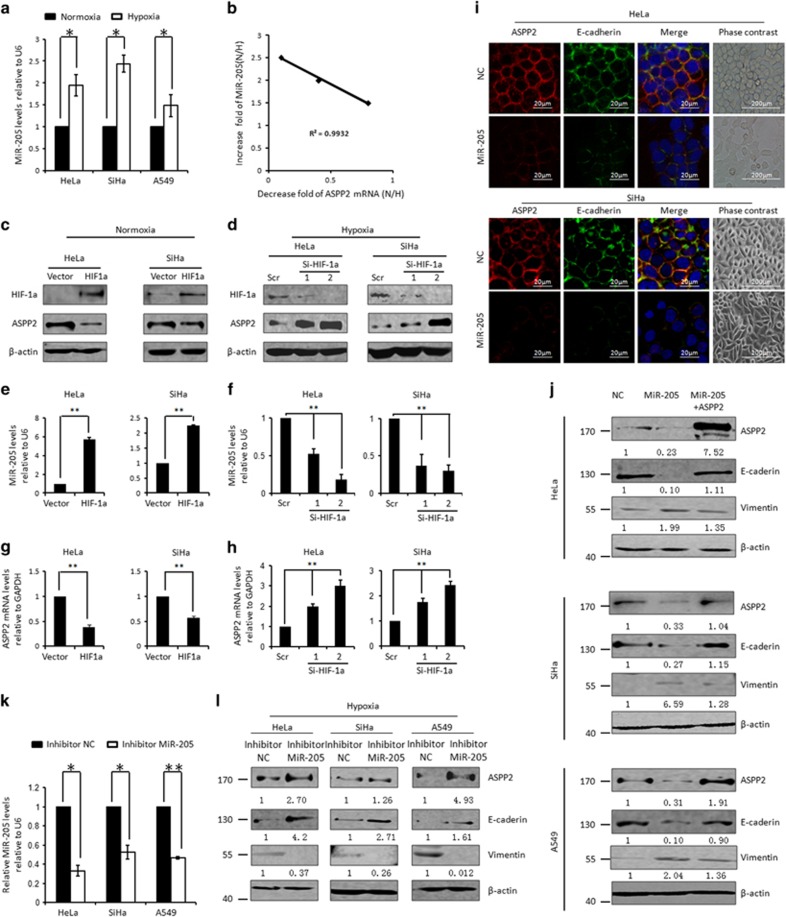
Hypoxia inhibits ASPP2 by the promoted MiR-205. (**a**) Real-time RT-PCR assay of MiR-205 under normoxia and hypoxia conditions. Histograms represent the mean±S.E.M. from three independent assays. (**b**) Linear correlation between decreased ASPP2 and increased MiR-205 was obtained in cancer cell lines (*r*^2^=0.9932, *P<*0.01). (**c**) WB analysis of HIF-1a and ASPP2 after ectopic expression of HIF-1a under normoxia conditions. *β*-Actin was used as a loading control. (**e** and **g**) Real-time RT-PCR analysis of MiR-205 (**e**) and ASPP2 (**g**) under the conditions as described in **c**. (**d**) WB analysis of HIF-1a and ASPP2 after preventing hypoxia-induced HIF-1a by transfection with two independent RNAi specifically targeting HIF-1a (Si-HIF-1a 1 and 2). *β*-Actin was used as a loading control. (**f** and** h**) Real-time RT-PCR analysis of MiR-205 (**f**) and ASPP2 (**h**) under the conditions as described in **d**. (**i**) Immunostaining analysis of E-cadherin/ASPP2 expression and localization after transfection with NC mimic and MIR-205 mimics. (**j**) WB analysis of ASPP2, E-cadherin and Vimentin after transfection with negative control, MiR-205 mimics or MiR-205 mimics+ASPP2 in HeLa, SiHa and A549 cells. (**k**) Real-time RT-PCR analysis of MiR-205 after transfection with NC inhibitors or MiR-205 inhibitors under hypoxia conditions. (**l**) WB analysis of ASPP2, E-cadherin and Vimentin under the conditions as described in **k**. *β*-Actin was used as a loading control. All histograms represent the mean±S.E.M. from three independent assays. *P<0.05; **P<0.01

We then asked whether MiR-205 is involved in ASPP2 inhibition under hypoxia conditions. First of all, the expression of MiR-205 was compared between cells cultured under the normoxia and hypoxia conditions. Remarkably, MiR-205 was increased in various cells under hypoxia conditions (44.4%, *P*<0.05 in Hela cells; 47.7%, *P*<0.05 in SiHa cells and 13.0% *P*<0.01 in A549 cells, Figure 3a). Interestingly, MiR-205 induction was linearly correlated with ASPP2 suppression (*r*^2^=0.9932, *P*<0.01, Figure 3b). In addition, accumulation of HIF-1a is the most critical adaptive response to hypoxia. Ectopic expression of HIF-1a promoted MiR-205 expression by 5.7-folds in HeLa and 2.2-folds in SiHa cells (*P*<0.01, Figures 3c and e), suggesting that hypoxia-induced MiR-205 expression is largely dependent on HIF-1a. Supportively, preventing HIF-1a accumulation by RNAi-mediated HIF-1a knocking down under hypoxia conditions resulted in a significant suppression of MiR-205 expression (*P*<0.01, Figures 3d and f). Importantly, both mRNA and proteins of ASPP2 exhibited a negative association with MiR-205 upon genetic modulation of HIF-1a (*P*<0.01, Figures 3g and h). These data together suggested that ASPP2 is subjected to MiR-205 regulation under hypoxia conditions. In support of this, ASPP2 was significantly suppressed by MiR-205 mimics (Figure 3i, top panels, Figure 3j and Supplementary Figure S5). By contrast, MiR-205 inhibitor efficiently repressed hypoxia-induced MiR-205 by >50%, as confirmed by a real-time RT-PCR assay (Figure 3k). ASPP2 was correspondingly restored by the same treatment (top panels, Figure 3l). Therefore, MiR-205 is one of critical physiological regulator of ASPP2 under hypoxia conditions.

Next, the role of MiR-205 in regulating EMT was investigated. First, the elevation of MiR-205 was parallel with E-cadherin suppression and Vimentin induction with hypoxia switch (Figures 2a, 3a and Supplementary Figure S5). In addition, arbitrarily introducing MiR-205 mimic under normoxia conditions led to E-cadherin suppression and Vimentin induction (Figure 3i and middle panels, Figure 3j). Meanwhile, cells lost the typical epithelial morphology and exhibited an elongated fibroblast-like phenotype (Figure 3i). Conversely, decreased E-cadherin and increased Vimentin were both rescued upon MiR-205 inhibitor treatment under hypoxia conditions (middle panels, Figure 3l). These data implicated that MiR-205, like its target ASPP2, participates in hypoxia-induced EMT process, and behave as an EMT promoter, which is opposite to the biological outcomes generated by ASPP2.

We further examined the contribution of ASPP2 in MiR-205-dependent EMT by ASPP2 rescue experiment. Remarkably, the ability of MiR-205 in regulating EMT as presented by E-cadherin suppression and Vimentin induction was largely abrogated by arbitrary expression of ASPP2 (Figure 3j). Taken these data together, MiR-205/ASPP2 axis is critical in hypoxia-induced EMT.

### MiR-205/ASPP2 axis promotes cell migration

**Figure 4:**
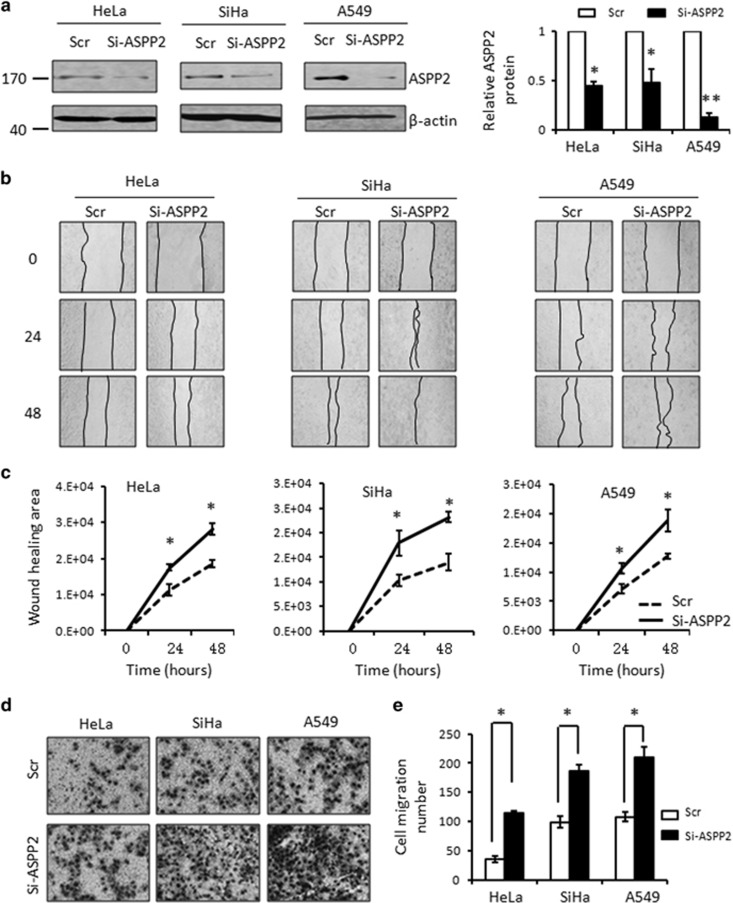
Inhibition ASPP2 promotes cell migration. (**a**) The efficiency of RNAi specifically targeting ASPP2 (Si-ASPP2) in comparison with scramble control (Scr) was confirmed by western blotting (left). *β*-Actin was used as a loading control. The bands were quantified using Image J software as normalized to *β*-actin (Right). (**b**) *In vitro* wound-healing analysis of the cells transfected with either Scr or Si-ASPP2 cells. Representative photographs at different time points of 24 and 48 h after making the wound were presented. (**c**) The plot represents the quantitative analysis of wound healing area from three independent experiments. Error bars, mean±S.E.M. (**d**) Migration rate was detected by a transwell assay in Scr and Si-ASPP2 cells. Representative photographs were presented. (**e**) The bar graph was the quantitative analysis of transwell assay. Error bars, mean±S.E.M. (*n*=3 independent experiments). *P<0.05, **P<0.01

Cells undergoing EMT generally exhibits elevated cell motility; therefore, we further examined the impact of MiR-205/ASPP2 axis on cell migration. ASPP2 was suppressed by RNAi (Si-ASPP2), which specifically targets ASPP2 mRNA degradation. ASPP2 was consistently decreased up to 60–90% as shown in Figure 4a in multiple cell lines, compared with the scramble controls (*P*<0.01). Cell motility was then evaluated by a wound-healing assay in cells with or without ASPP2 silencing. As shown in Figures 4b and c, inhibiting ASPP2 significantly increased cell migration rates. The difference became significant 24 h after making the wound and turned more evident over time (*P*<0.05, Figures 4b and c). The migration alternation was further confirmed by transwell assays (Figures 4d and e). Similarly, cells with ASPP2 silencing migrate more efficiently through the micropore wells than the cells treated with scramble controls. The increased migration rate ranged from two- to three-folds in a cell type-dependent manner (*P*<0.05, Figures 4d and e).

**Figure 5:**
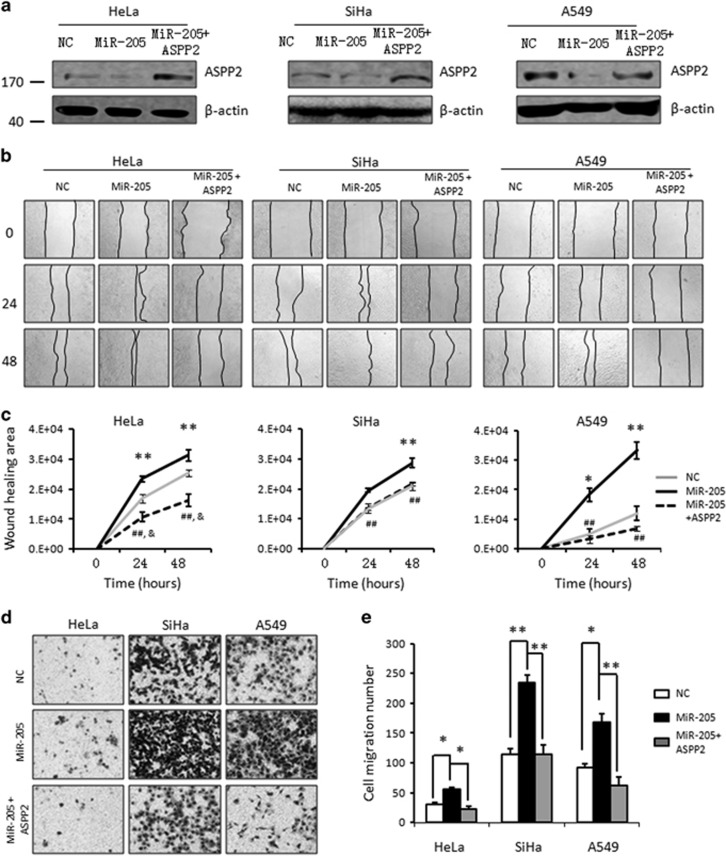
MiR-205-mediated ASPP2 suppression promotes cell migration. (**a**) WB analysis of ASPP2 after transfection cells with control, MiR-205 mimics and MiR-205 mimic+ASPP2. *β*-Actin was used as a loading control. (**b**) *In vitro* wound-healing analysis in negative control, MiR-205 or MiR105+ASPP2 cells. Representative photographs at different time points after making the wound were presented. (**c**) Wound-healing areas over time were quantified by using Image J. Error bars, mean±S.E.M. (*n*=3 independent experiments). *P<0.05; **P<0.01, miR-205 *versus* NC; ^&^*P*<0.05,miR-205+ASPP2 *versus* NC; ^##^*P*<0.01,miR-205+ASPP2 *versus* miR-205 mimics. (**d**) Migration rate was also detected by a transwell assay in negative control, MiR-205 or MiR105+ASPP2 cells. Representative photographs were presented. (**e**) The bar graph was the quantitative analysis of transwell assay. Error bars, mean±S.E.M. (*n*=3 independent experiments). *P<0.05, **P<0.01

The ability of MiR-205 in regulating cell migration was also examined in the same set of cell models. MiR-205 overexpression inhibited ASPP2 expression (Figure 5a). Similar to ASPP2 silencing, these cells with MiR-205 overexpression exhibited an increased migration rate revealed by both wound-healing assays and transwell assays (Figures 5b and e). Remarkably, re-expression of ASPP2 prevented the enhanced cell migration induced by MiR-205 (Figures 5b and e), suggesting ASPP2 is one of major targets of MiR-205 that confers to MiR-205-promoted cell migration.

### MiR-205/ASPP2 axis promotes cell growth both *in vitro* and *in vivo*

**Figure 6:**
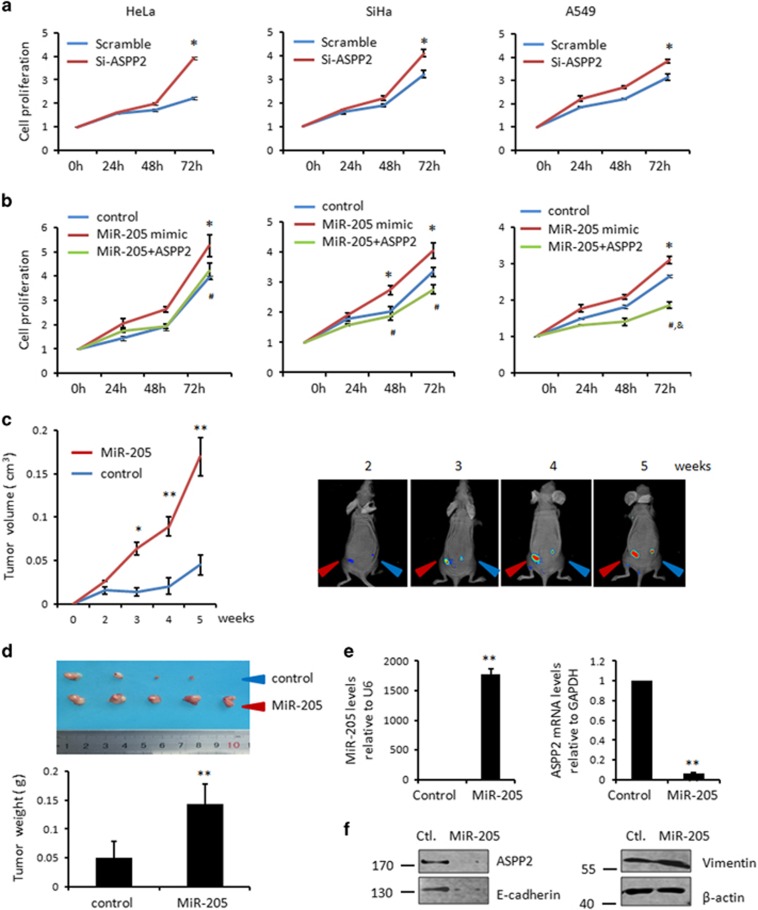
MiR-205/ASPP2 axis promotes cell growth both *in vitro* and *in vivo*. (**a**) Cell proliferation was determined in triplicates at 24-h intervals in the cells transfected with Scr or Si-ASPP2 by MTT assays. Error bars, mean±S.E.M. (*n*=3 independent experiments). *P<0.05. (**b**) Cell proliferation was determined by a similar MTT assay as described in **a** in NC, MiR-205 mimics or MiR-205 mimic+ASPP2 transfected cells. Error bars, mean±S.E.M. (*n*=3 independent experiments). *P<0.05, miR-205 *versus* NC; ^&^*P*<0.05, miR-205+ASPP2 *versus* NC; ^#^*P*<0.05, miR-205+ASPP2 *versus* miR-205 mimics. (**c**) Tumor growth in nude mice subcutaneously injected into flanks with SiHa/DsRed control and SiHa/DsRed+MiR-205. Representative photographs of the tumors at different time after inoculation with either SiHa/DsRed control or SiHa/DsRed+MiR-205 cells (right). Tumor volume was measure according to materials and methods. Data are presented as means±S.D. (*n*=5 per group) (left). *P<0.05, **P<0.01. (**d**) Photographs of the dissected tumors of either SiHa/DsRed control or SiHa/DsRed+MiR-205 cells (upper). Average of tumor weight was presented in the bar graph. Data are presented as means±S.D. (*n*=5 per group) (bottom). **P<0.01. (**e**) Real-time analysis of MiR-205 and ASPP2 in the representative tumors. Histograms represent the mean±S.E.M. from three independent assays. **P<0.01. (**f**) WB analysis of ASPP2, E-cadherin and Vimentin in the representative tumors. *β*-Actin was used as a loading control

As cell–cell contact disruption can also promote cell proliferation, we further asked whether MiR-205/ASPP2 also affect cell proliferation. The survival rates of cervical and lung cancer cells were significantly enhanced in ASPP2 silenced cells. This increased proliferation appeared at 24 h in SiHa and A549 cells, and at 48 h in Hela cells, which turned more obvious over time. Statistic significance were obtained at 72-h cultures in all three cells studied (*P<*0.05, Figure 6a). By contrast, MiR-205 influenced cell proliferation in an opposite way from ASPP2, which markedly promoted cell proliferation (*P<*0.05, Figure 6b). Conversely, MiR-205 inhibitor significantly suppressed MiR-205 expression under hypoxia conditions and simultaneously inhibited cell proliferation rates in both HeLa and SiHa cells (*P*<0.05, Supplementary Figure S6). Importantly, recovery ASPP2 expression successfully inhibited the MiR-205-mediated cell growth (Figure 6b). These data suggested that ASPP2 is an important downstream target of MiR-205 to promote cell proliferation.

The influence of MiR-205/ASPP2 on cell survival was further confirmed *in vivo* by using xenograft mice models. SiHa cells stably expressing DsRed control or DsRed+MiR-205 by lentivirus expression system were injected subcutaneously in nude mice. To minimize the potential variation generated by the nature of individual nude mice, the same number of SiHa/DsRed control and SiHa/DsRed+MiR-205 cells were paired and injected into one nude mouse at symmetrical subcutaneous regions, respectively (Figure 6c). The DsRed fluorescence signal was monitored by the *in vivo* imagining system weekly. The tumor burden became evident in both SiHa sublines 2 weeks after injection. Since then, MiR-205 overexpression xenograft showed a more markedly increased growth rate than the control. The difference in tumor size between the two groups became statistic significant 3 weeks after injection (*P<*0.05) and turned more evident over time (*P<*0.01, Figure 6c). At the end of experiments, the mice were dissected, and the tumor weight was measured immediately. As shown in Figure 6d, the tumor weight was significantly increased with MiR-205 overexpression cells (*P<*0.01, Figure 6d). The overexpression of MiR-205 was confirmed in the dissected xenograft. The downregulation of ASPP2 was also confirmed at mRNA levels by real-time RT-PCR (*P*<0.01, Figure 6e) and at protein levels by western blotting (Figure 6f). Furthermore, the decreased E-cadherin and increased Vimentin were correspondingly observed in the representative samples of SiHa-DsRed+MiR-205 cells as shown in the Figure 6f. These data together suggest that, consistent with the *in vitro* data described above, MiR-205/ASPP2 axis promotes cell growth *in vivo*.

## Discussion

**Figure 7:**
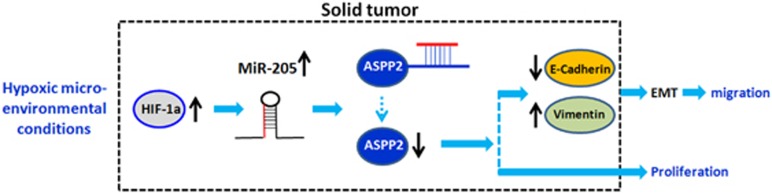
Proposed model of MiR-205 upregulation involved in promoting EMT process via targeting ASPP2. Under hypoxia conditions, HIF-1a is accumulated. MiR-205 is subsequently increased, which then targets ASPP2 by interacting directly with the conserved 3′-UTR sites of ASPP2. ASPP2 expression is suppressed by MiR-205 and therefore confers to the promoted MT process via inhibiting E-cadherin. Meanwhile, MiR-205/ASPP2 axis also contributes to the elevated cell proliferation in both cell types tested in this study, cervical and lung cancers

Here we identified that ASPP2 is novel tumor-suppressor target of MiR-205 and has key roles in mediating MiR-205-induced EMT, which provides important molecular explanations for the not yet well-defined oncogenic functions of MiR-205, particularly in promoting invasion and metastasis. Furthermore, we also found that the newly identified MiR-205/ASPP2 axis can be modulated by microenvironmental hypoxia, implicating a critical impact of MiR-205/ASPP2 on promoting cancer progress in the hypoxia niche of solid tumors (Figure 7).

Downregulation of MiR-205 has been observed in a panel of cancers, such as breast, prostate, bladder and gliomas and head and neck, pointing to a tumor-suppressor activity of MiR-205.^14, 34, 35, 36, 37, 38^ Indeed, most of its targets identified so far are oncogenes (e.g., ErbB3, VEGF-A, ZEB1/2, ZEB1/2, ΔNp63*α*, SFK and PKC*ɛ*).^18^ Simultaneously, however, evidence for an oncogenic role of MiR-205 also began to accumulate. For example, Xu *et al.* found that MiR-205 confers to the elevated radiation resistance of human nasopharyngeal carcinoma by targeting PTEN.^20^ The MiR-205/PTEN axis has later been reported also to promote cell growth in lung and endometrial cancer.^19, 39, 40^ In addition, overexpression of MiR-205 is associated with the suppression of another two targets, CYR61 and CTGF, in cervical cancers and SHIP-2 in squamous cell carcinoma.^21, 41^ More recently, MiR-205 was also reported to promote cell migration and invasion in normal keratinocytes, ovarian and cervical cancer cells,^21, 22, 42^ which is in support of our results obtained in this study. Moreover, we further identified that this oncogenic capability of MiR-205 is possibly attributed to its activity in promoting EMT. This result seems controversial with the well-established concept that MiR-205 expression inhibits, rather than promotes, EMT program by targeting ZEB1/2, leading to an increased E-cadherin in breast, bladder and prostate cancers.^14, 43, 44^ Nevertheless, despite detailed mechanisms remain unknown, Li *et al.*^45^ reported that MiR-205 expression is associated with the E-cadherin suppression during the development of extra embryonic endoderm, which is in consistent with our results. So, our data together with others suggested that MiR-205 may have a double-edged sword effect on EMT. One potential explanation is that MiRNAs can target multiple genes, therefore, the biological outcomes of MiRNA activation may change with cell content and stimulus. Yu *et al.*^41^ proposed that MiR-184 can interfere with the binding between MiR-205 and its target SHIP-2 mRNA. We can not exclude the possibility that other similar MiRNAs also exist in cervical and lung cancer cells, which may be involved in the regulation of the selectivity of MiR-205 in binding with its potential targets. An alternative explanation is that MiRNA fulfill its task via binding with 3′-UTR region of target mRNA. However, the expression of tumor suppressor can be regulated by numbers mechanisms, such as gene deletion or epigenetic promoter methylation and histone modification. So, if those tumor-suppressor targets of MiR-205, such as ASPP2,^46^ are suppressed by the above mentioned mechanisms, no targeting mRNA will be available for MiR-205 to bind. Instead, MiR-205 may selectively regulate its oncogene target, such as Zeb1, which is normally highly expressed in cancers and thus lead to promoted EMT in malignancies, such as breast cancers.

In contrast to the anti-EMT mechanism of MiR-205, the exact mechanisms underlying the pro-EMT activities of MiR-205 remain largely unknown. Here, we found that ASPP2 is a direct target of MiR-205. The inverse association between ASPP2 and MiR-205 is not only validated by transfection with either MiR-205 mimic or inhibitors, and also observed in cervical tissues. Importantly, ASPP2 suppression produces a similar biological outcome to MiR-205 in regulating EMT. Critically, arbitrary expression of ASPP2 can prevent MiR-205-induced EMT. These data together suggest that ASPP2 is a key target of MiR-205 to promote EMT.

Initially identified as a p53 activator, ASPP2 has been recently found to form complex with tight junction component Par-3 and thus has important roles in maintaining epithelial polarity.^30, 31^ Interestingly, tight junctional components are important targets for MiRNA-regulated EMT progress, as demonstrated in a microarray experiment.^47^ This is also in keep with another report by Chung *et al.*^48^ that there are 11 tight junction proteins among 24 significant validated targets of MiR-205 in mammalian bladder urothelial cells. These data are in support of our findings that ASPP2 is a key target of MiR-205 in regulating EMT. Wang *et al.*^33^ provided solid evidence that ASPP2 inhibits EMT by stabilization of *β*-catenin–E-cadherin complex at cell–cell junctions and subsequent inhibition of Wnt signaling transduction pathway. Here we found that ASPP2 is mainly localized at cell–cell junctions. MiR-205 suppresses ASPP2 by inhibiting its transcription and protein expression and thus depleted its function at junction.

It is also noteworthy that hypoxia is a hallmark of solid cancer.^49^ Cancer cells gain the capability to adapt to hypoxia via modulating gene expression patterns by utilizing different strategies.^50^ Hypoxia induces upregulation of MiR-205 and subsequent inhibition of ASPP2, resulting in EMT, migration and proliferation, which is thus an adaptative and protective response to hypoxia. The newly identified pro-EMT activity of MiR-205 indicated that MiR-205 inhibitor may be potentially used to treat cervical cancer and lung cancer and counteract with hypoxia-induced malignant growth. It will be interesting to further test this hypothesis *in vivo* by generating well-controlled hypoxic xenografts. It is also worth noting that MiR-205 has been reported to be suppressed under hypoxia conditions in prostate cancer and renal cell carcinoma cells.^51, 52^ These reports together with ours support previous idea raised by Camps *et al.*^53^ By a genome-wide study, they found that MiRNA expression can be regulated by hypoxia in a tissue-specific manner. This may be due to the complex regulatory machineries of MiRNAs. The underlying mechanisms of hypoxia-regulated MiR-205 expression warrant further examination.

To our knowledge, despite MiRNAs have been widely accepted as an important regulator of gene expression, their roles in regulating ASPP2 remains unknown. For the first time, we provided important information on the epigenetic regulation of ASPP2 by MiRNAs, MiR-205. Of note is that dysfunction of ASPP2 under hypoxia condition may be fulfilled by various mechanisms in addition to MiR-205-mediated ASPP2 silencing. For example, Kim *et al.*^54^ recently found that ASPP2 can be inhibited by Siah2-induced proteasome degradation under hypoxia conditions.

Taken together, hypoxia/MiR-205/ASPP2/EMT is a newly identified mechanism for cancer progression. Inhibiting MiR-205 or rescue ASPP2 are potential therapeutic strategies to inhibit the metastasis of cervical and lung cancers.

## Materials and Methods

### Tissue samples

Samples of six cervical cancer and the paired normal controls were obtained from the First Affiliated Hospital, Harbin Medical University. All samples were obtained with approval from the institutional ethics committee.

### Cell lines and culture conditions

The human cervical cancer cell lines (Hela and SiHa) and human lung cancer cell line A549 were obtained from the American Type Culture Collection (ATCC, Manassas, VA, USA). The cells were cultured in DMEM medium supplemented with 10% fetal bovine serum (FBS). Cells were incubated at 37 °C incubator in an atmosphere of 5% CO_2_. For hypoxia treatment, cells were cultured in an atmosphere of 2% O_2_, 5% CO_2_ at 37 °C in a hypoxia incubator for the indicated period of time. All cell lines were authenticated and characterized by the supplier. The cells were expanded immediately and multiple aliquots were cryopreserved. All cell lines have never been passaged longer than 3 months and the cell lines were characterized by Genetic Testing Biotechnology Corporation (Suzhou, China) using short tandem repeat markers.

### RNA extraction and quantitative RT-PCR

Total RNA was isolated from cells with Trizol (Invitrogen, Carlsbad, CA, USA) following the manufacturer's protocol. In all, 1 *μ*g total RNA was reverse transcribed using a PrimeScript reverse transcription (RT) reagent kit in the presence of gDNA Eraser. cDNA synthesis was suggested by the kit protocol. Reverse transcription was performed for 15 min (min) at 37 °C, 85 °C for 5 s and finally at 4 °C forever. After the RT reaction, the cDNA was diluted 10-fold and performed the quantitative RT-PCR using SYBR Premix Ex Tag II. The conditions were as follows: 95 °C for 30 s, 40 cycles of 95 °C for 5 s, 58 °C for 34 s and last stage at 95 °C for 15 s, 60 °C for 1 min, 95 °C for 15 s by using an Applied Biosystems 7500 real-time PCR system (Applied Biosystems, Branchburg, NJ, USA). mRNA levels were calculated according to the Ct value. The housekeeping gene GAPDH was used as an internal control. The primer sequences used in this study are listed below: ASPP2 forward 5′-GAAGACTCGGTGAGCATGCG-3′, reverse 5′-GCGATACGCTCTGAGCCAGT-3′ VEGF forward 5′-GAATCATCACGAAGTGGTGAAGT-3′, reverse 5′-GTTGGACTCCTCAGTGGGC-3′ and GAPDH forward 5′-CGACCACTTTGTCAAGCTCA-3′, reverse 5′-ACTGAGTGTGGCAGGGACTC-3′.

### Western blot

Proteins were extracted from cells using urea buffer (2 M Thiourea, 4%CHAPS, 40 mM Tris-Base, 40 mM DTT, 2%Pharmalyte). Equal amount of proteins were separated at sodium dodecyl sulfate-polycylamide gel electrophoresis (SDS-PAGE), and then transferred to PVDF membranes using the cold transfer buffer. Membranes were blocked with 5% non-fat milk in TBS-T for 1 h at room temperature, and subjected to corresponding primary antibody at 4 °C overnight. Antibodies used were list as bellow: ASPP2 (Sigma-Aldrich, St Louis, MO, USA, 1 : 2000), *β*-actin (Sigma-Aldrich, 1 : 2000), HIF-1*α* (Abcam, Cambridge, UK, 1 : 750), Vimentin (Santa Cruz, CA, USA, 1 : 2000), E-cadherin (Santa Cruz, 1 : 2000) and GAPDH (Sigma-Aldrich, 1 : 2000). Secondary antibodies, goat anti-mouse-HRP and goat anti-rabbit-HRP, were diluted at 1 : 2000. Signals were visualized by ECL. Membrane was then ready for scanning by Image studio system (ECL, LI-COR, Lincoln, Georgia, USA). Protein quantification was conducted by Image J software (National Institutes of Health, Bethesda, MD, USA). The gray values of protein were achieved as gray level of protein band/gray level of loading control.

### MTT

Cell proliferation was detected by the colorimetric MTT assay. The cell lines were plated in 96-well plates. After 0-h, 24-h, 48-h and 72-h incubation, cells were treated with MTT solution at the concentration of 5 mg/ml for 4 h at 37 °C. The MTT was discarded and formazan was dissolved with 100 *μ*l DMSO. Finally, the absorbance at 490 nm was determined using Microplate Reader (Tecan Austria GmbH 5082, Grodig, Austria).

### Luciferase assay

The 3′-UTR sequence of ASPP2 was amplified from genomic DNA derived from HEK293 cells by PCR. The PCR product was then inserted into pMIR-REPORT vector (Applied Biosystems). According to the binding sites of MiR-205 on 3′-UTR region of ASPP2, the mutant (MUT) pMIR-ASPP2-Report was generated by using mutagenesis kit according to the manufacturer's introduction using wild type (WT) pMIR-ASPP2-Report as a template (Stratagene, TransGen, Beijing, China). The cells were seeded into 24-well plates by 3 × 10^4^ cells per well and then transfected with a mixture of 15 ng pRL-TK Renilla luciferase, 40 pmol MiR-205 mimic or negative control and 300 ng WT or MUT pMIR-ASPP2-Report using Lipofetamine 2000 (Invitrogen). After 48 h, the cells were harvested and subjected to a assay by using the Dual Luciferase Reporter Assay system (Promega, Madison, WI, USA). The relative luciferase activities were normalized with the Renilla luciferase activities.

### MiRNAs isolation and Taqman quantitative RT-PCR

MiRNAs were isolated from cells or paired cervical tissues by using the mirVana miRNA isolation kits (Applied Biosystems) following the manufacturer's protocol. In all, 15 ng mature miRNAs was used to synthesis cDNA by Taqman MicroRNA Reverse Transcription kit (Applied Biosystems). The cDNA was diluted 10-fold and the quantitative RT-PCR using Taqman MicroRNA Assay kit (Applied Biosystems) was performed. Relative miRNA expression levels were calculated using rRNA U6 as an internal control.

### Xenografted tumor model *in vivo*

All animal experiments were performed according to protocols approved by the Institutional Animal Care and Use Committee. The female nude mice between 4 and 5 weeks were purchased from Beijing HFK Bioscience Co., Ltd (Beijing, China). SiHa cells infected with lentivirus constructs of LV10-NC or LV10-MiR-205 using Lipofectamine 2000 (Invitrogen). SiHa stable lines (1 × 10^7^) were transplanted subcutaneously into either side of flank of the same female nude mice. The tumor volumes were measured every week and calculated as length × width^2^ × 0.5. After 5 weeks after injection, the mice were anesthetized and killed. The tumor were carefully removed, photographed and weighed. The removed tumors were immediately stored in the liquid nitrogen. RNA and protein were isolated and subjected to the qPCR and western blotting assays.

### Wound-healing assay

The 1 × 10^5^ cells were seeded in 24-well plate and were incubated at 37 °C in 5% CO_2_ for 24 h and subjected to serum starvation for additional 24 h before making the wound. To avoid cell proliferation, cells were maintained in the serum-free medium throughout of the experiment. An approximately 0.4–0.5 mm line was scraped using the fine end of sterile pipette tips. Then the plate was washed with 1 × PBS for three times and cultured with serum-free medium for the indicated period of time. The images were captured by inverted microscope. The images were then analyzed using Image J software.

### Transwell assay

In all, 5 × 10^4^ cancer cells were suspended in 150 *μ*l serum-free medium added to the upper chamber and a total of 600 *μ*l medium with 10% serum was added to the bottom chamber as a chemoattractant. The 24-well Boyden chamber (8 *μ*m; Corning, NY, USA) were incubated at 37 °C for 48 h. After wiping off the non-migrating cells in the upper chamber attentively with cotton swabs, the cells on the polycarbonate membranes were fixed with 4% paraformaldehyde and stained with 0.5% crystal violet (Sigma, St. Louis, MO, USA). The cells were counted in five random fields on each membrane.

### Immunofluorescence assay

Cells grown on cover slips in a 24-well plate were fixed in 4% paraformaldehyde solution for 20 min after three washes in pre-warmed 1 × PBS. After another round of washing in 1 × PBS, cells were permeabilised with 1% Triton X-100 solution on ice for 4 min. The permeabilisation solution was then removed by adding 1 × PBS, following which cells were blocked in 1% fish gelatin (G7765, Sigma-Aldrich) for 1 h. Incubation with the appropriate diluted primary antibody was performed over night at 4°C, followed by three washes in 1 × PBS for 5 min each. Antibodies used included anti-ASPP2 (Sigma-Aldrich, 1 : 100), anti-E-cadherin (Abcam,1 : 100) and anti-v5 (Serotec, 1 : 100). Cells were then incubated with the fluorescently labeled secondary antibody for 1 h. Typically, these secondary antibodies were used at 1 : 400 dilutions in blocking solution. The secondaries were replaced with 1 × PBS and rinsed in H_2_O before being mounted by Mowiol (Sigma, St. Louis, MO, USA) onto slides. Images were captured by confocal microscopy.

### Statistical analysis

Data are expressed as the mean±S.D. Student's *t*-test and one-way ANOVA analysis were performed to analysis the date using GraphPad software (La Jolla, CA, USA). Each experiment was performed at least three times. A value of *P<*0.05 was considered significant.
