# Supplementary material for: Upregulation of MiR-205 under hypoxia promotes epithelial–mesenchymal transition by targeting ASPP2
Source: Cell Death Dis. 2016 Dec 8;7(12):e2517–. doi: 10.1038/cddis.2016.412 (PMC5261019; doi:10.1038/cddis.2016.412)
Supplement: Supplementary Figure Legends [file cddis2016412x1.docx]

**Up-regulation of MiR-205 under hypoxia promotes epithelial-mesenchymal transition by targeting ASPP2**

Xingwen Wang^1,2^, Miao Yu^3^, Kunming Zhao^1^, Mengmeng He^1^, Wenjie Ge^1,2^, Yuhui Sun^4,*^, Yihua Wang^5^, Haizhu Sun^f^, and Ying Hu^1,2,*^

^1^ School of Life Science and Technology, Harbin Institute of Technology

^2^  Shenzhen Graduate School of Harbin Institute of Technology

^3^ School of Chemical Engineering and Technology, Harbin Institute of Technology

^4^ The first affiliated hospital, Harbin Medical University

^5^ Center of Biological Sciences, University of Southampton

^6^ The second affiliated hospital, Harbin Medical University

**Supplementary Figure S1. ASPP2 is a potential target of MiR-205. (a) The potential miRNA binding sites located at 3’-UTR regions of ASPP2 were predicted by the commonly cited bioinformatic tools such as microRNA.org, TargetScan4, miRBase, PicTar and miRanda. Five targets (MiR-205, 221, 222, 144 and 139) were predicted by at least four of above databases. (b) Western blotting (WB) and real-time RT-PCR analysis of ASPP2 expression in 293T cells transfected with the predicted miRNA (MiR-205, 221, 222, 144 and 139). β-actin was used as loading controls in WB assays. (c) Real-time RT-PCR analysis of ASPP2 expression in RC-1 cells transfected with MiR-205 mimics or MiR-205 inhibitor. Error bars, mean ± SEM (n = 3 independent experiments). *, *P<*0.05, **, *P<*0.01.**

**Supplementary Figure S2. MiR-205 expression is largely modulated by MiR205 mimics and inhibitors. Up- or down-regulation of MiR-205 expression was confirmed by Real-time RT-PCR analysis in HeLa, SiHa, A549 and 293T cells after MiR-205 mimics (a) or MiR-205 inhibitors (b) transfection. Error bars, mean ± SEM (n = 3 independent experiments). **, *P<*0.01.**

**Supplementary Figure S3. VEGF is induced under hypoxia conditions. Real-time RT-PCR analysis of well-known transcriptional target of HIF-1a, VEGF. Error bars, mean ± SEM (n = 3 independent experiments). *, *P<*0.05, **, *P<*0.01.**

**Supplementary Figure S4. E-cadherin and ASPP2 are down-regulated by hypoxia. The bar graph was the quantitative analysis of immunostaining results of ASPP2 and E-cadherin expression in HeLa (a) and SiHa (b) cells under normoxia and hypoxia conditions (related to Figure 2c) Error bars, mean ± SEM (n = 3 independent experiments). *, *P<*0.05, **, *P<*0.01.**

**Supplementary Figure S5. E-cadherin and ASPP2 are down-regulated by MiR-205 mimics. The bar graph was the quantitative analysis of immunostaining results of ASPP2 and E-cadherin in HeLa (a) and SiHa (b) cells after transfected with MiR-205 mimics or NC mimics (related to Figure 3i). Error bars, mean ± SEM (n = 3 independent experiments). *, *P<*0.05, **, *P<*0.01.**

**Supplementary Figure S6. MiR-205 inhibitors suppress cell growth under hypoxia conditions. (a,b) MiR-205 inhibitors-mediated MiR-205 suppression under hypoxia conditions was confirmed by real-time RT-PCR assay in HeLa (a) and SiHa (b). Error bars, mean ± SEM (n = 3 independent experiments). **, *P<*0.01. (c,d) Cell proliferation was determined by MTT assays in triplicates at 24 hours intervals in HeLa (c) and SiHa (d) cells with the same treatment as described in S6a and S6b. Error bars, mean ± SEM (n =3 independent experiments). *, P<0.05. (e,f) WB analysis of p21 expression in HeLa (e) and SiHa (f) cells with the same treatment as described in S6a and S6b. β-actin was used as a loading control.**
