# Supplementary figures and images for: Upregulation of MiR-205 under hypoxia promotes epithelial–mesenchymal transition by targeting ASPP2
Source: Cell Death Dis. 2016 Dec 8;7(12):e2517–. doi: 10.1038/cddis.2016.412 (PMC5261019; doi:10.1038/cddis.2016.412)

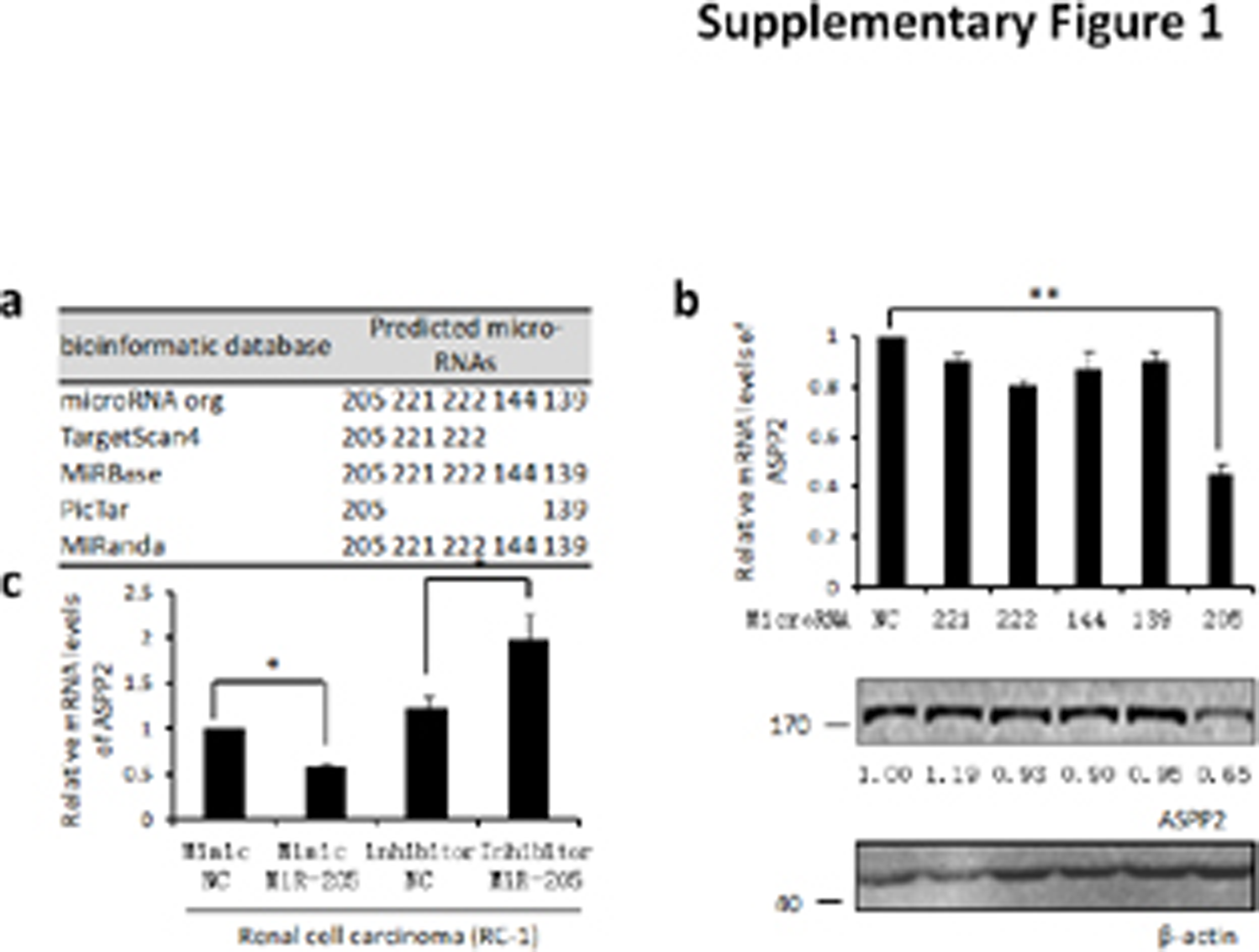

Supplement: Supplementary Figure S1 [file cddis2016412x2.tif]

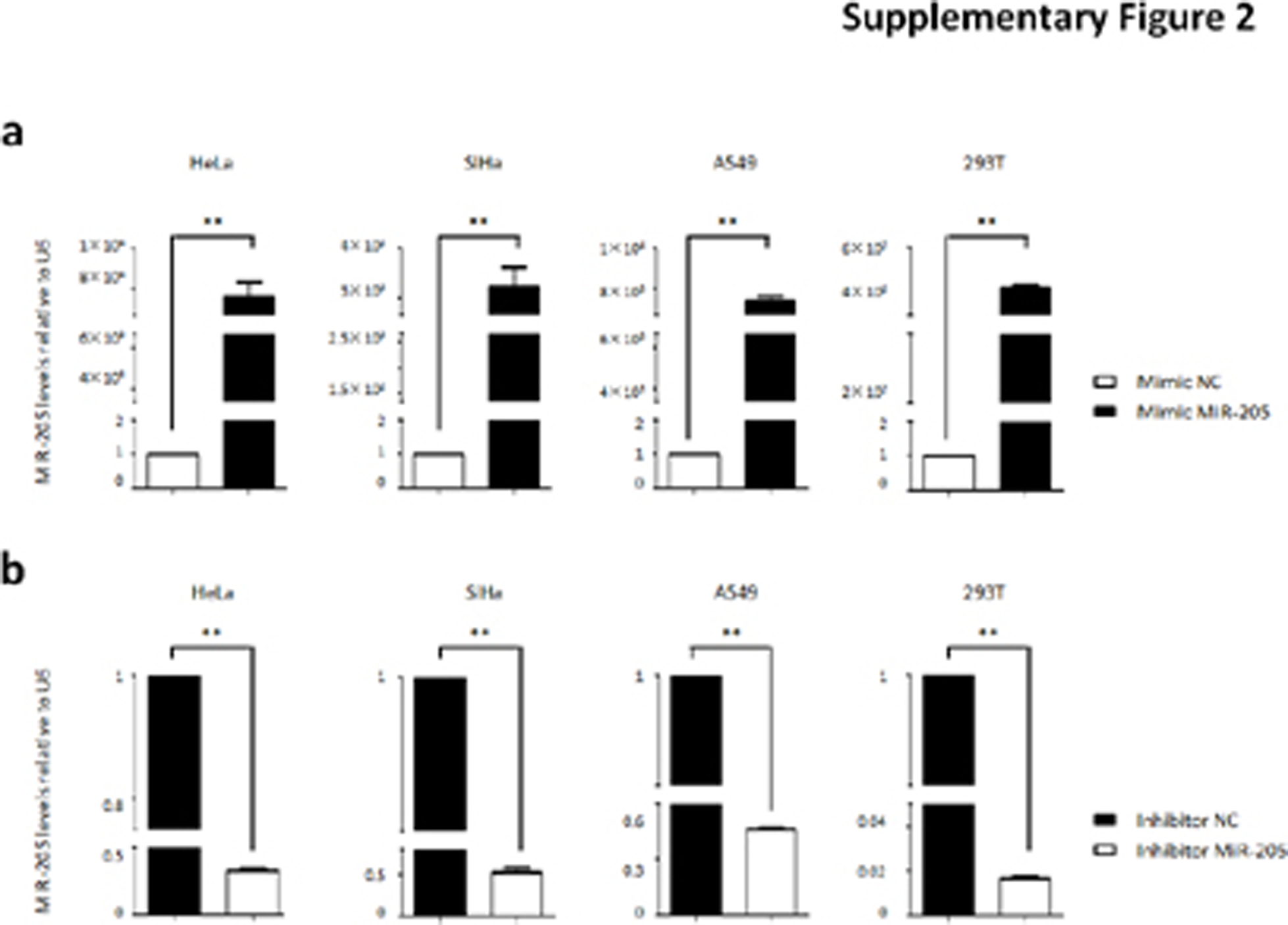

Supplement: Supplementary Figure S2 [file cddis2016412x3.tif]

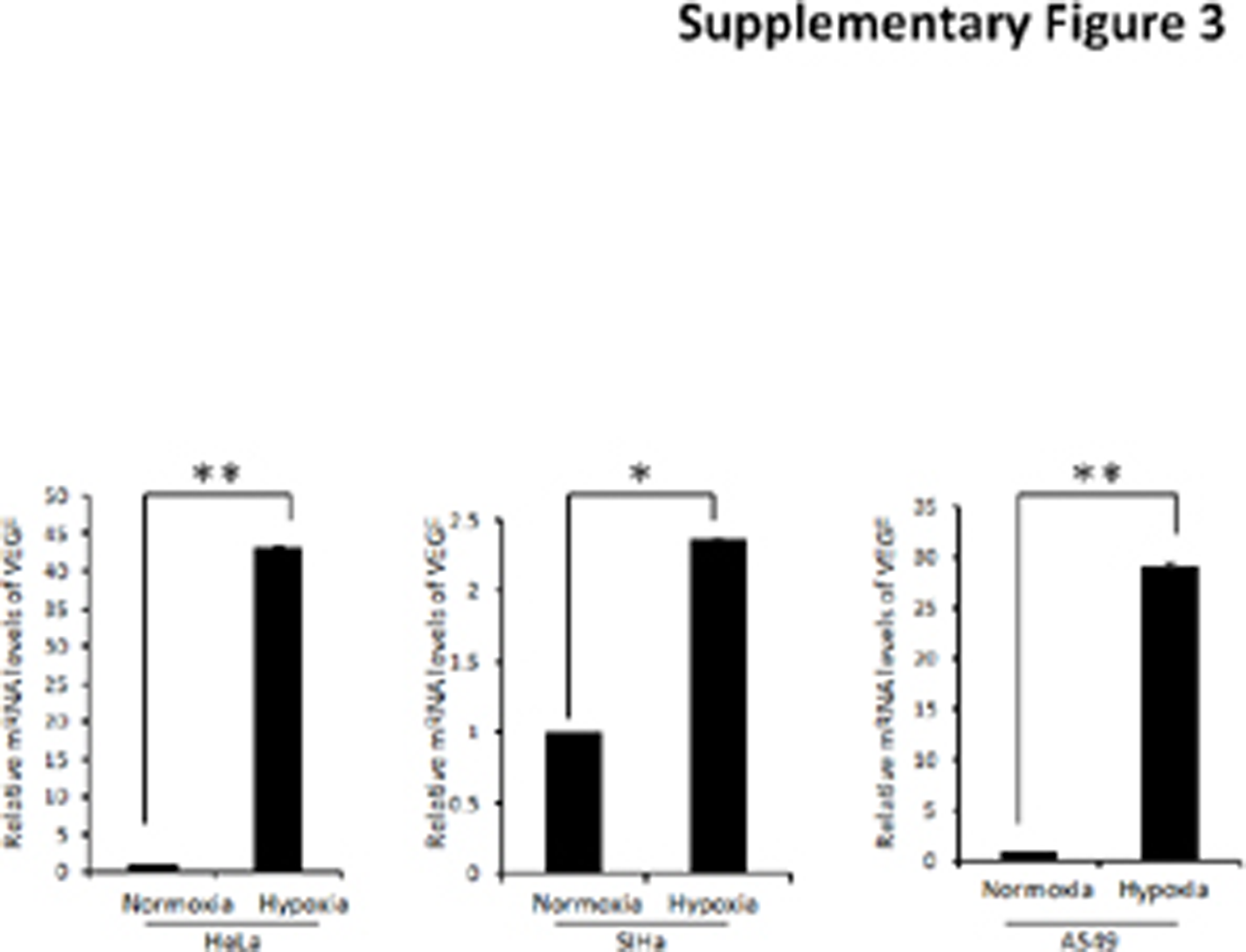

Supplement: Supplementary Figure S3 [file cddis2016412x4.tif]

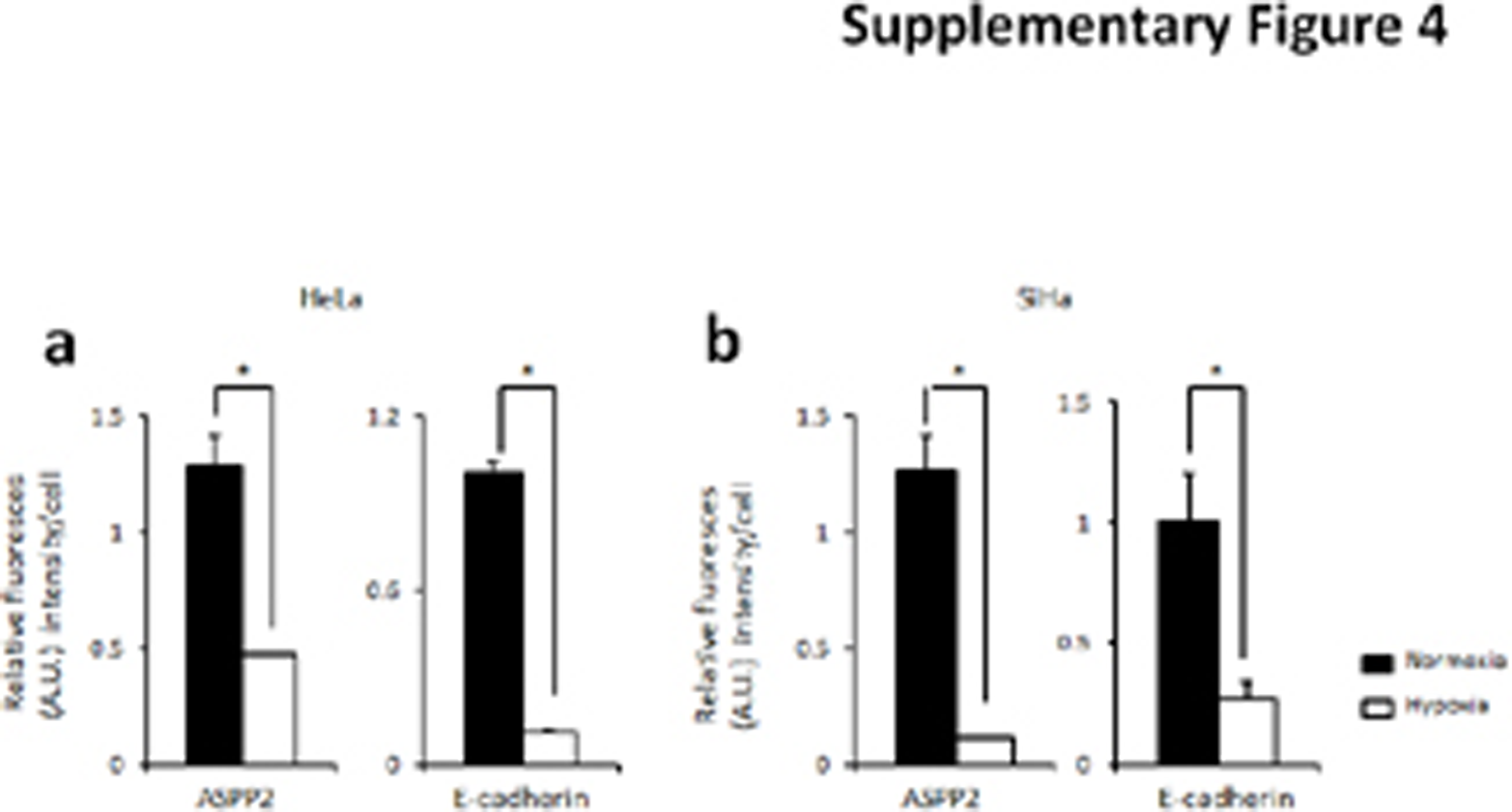

Supplement: Supplementary Figure S4 [file cddis2016412x5.tif]

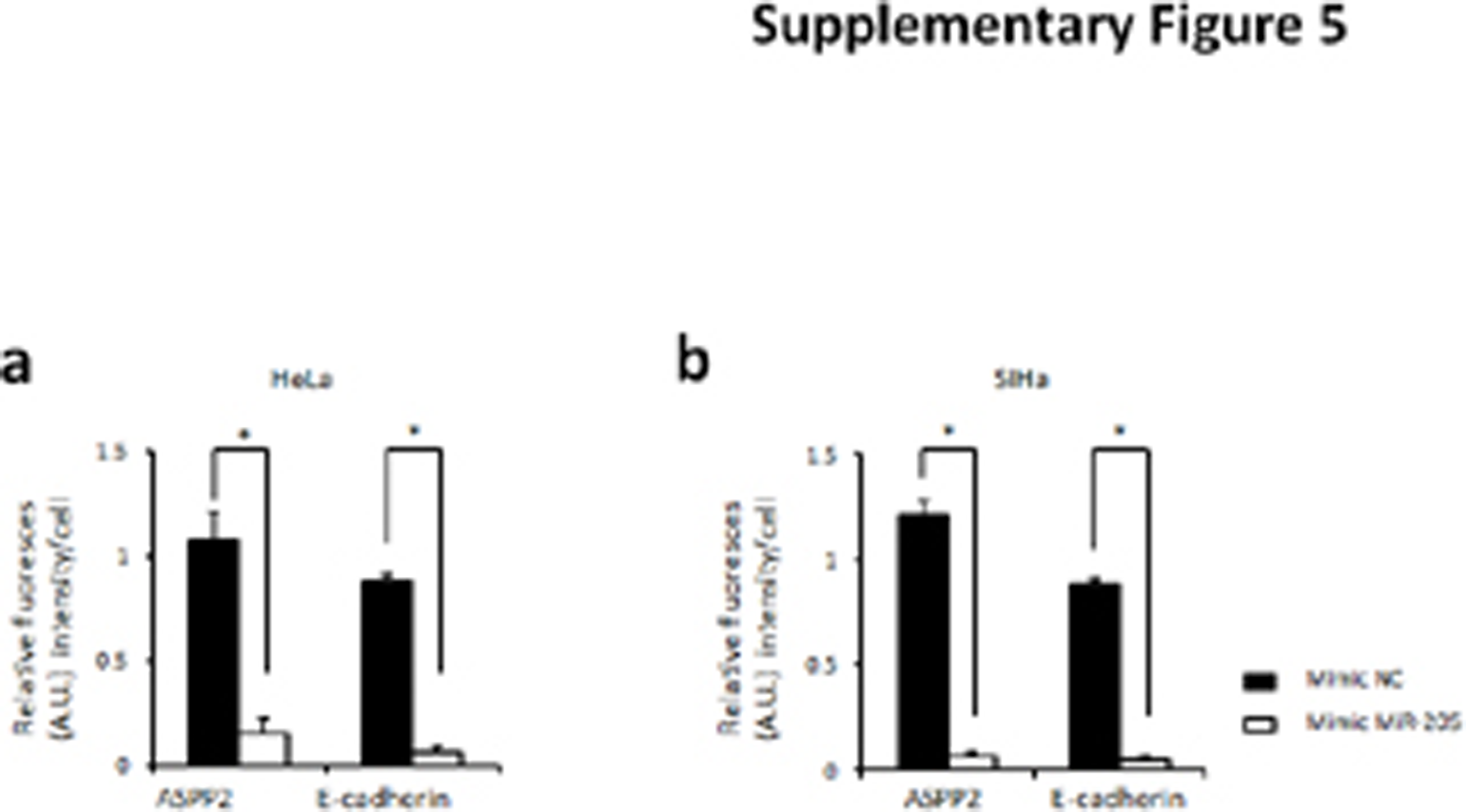

Supplement: Supplementary Figure S5 [file cddis2016412x6.tif]

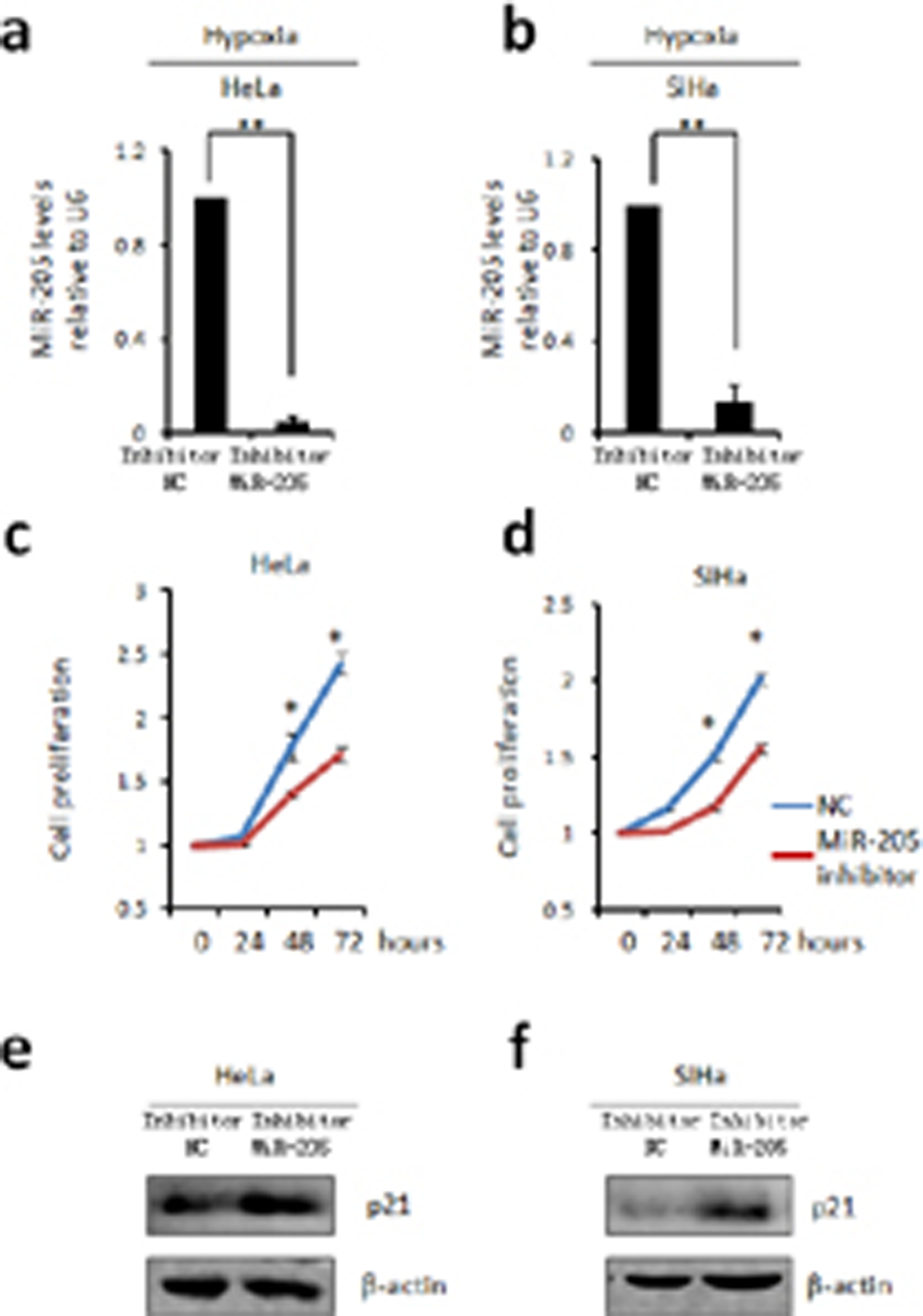

Supplement: Supplementary Figure S6 [file cddis2016412x7.tif]
